# Influence of habitat heterogeneity on anuran diversity in Restinga landscapes of the Parnaíba River delta, northeastern Brazil

**DOI:** 10.3897/zookeys.757.21900

**Published:** 2018-05-10

**Authors:** Kássio C. Araújo, Anderson Guzzi, Robson W. Ávila

**Affiliations:** 1 Programa de Pós-Graduação em Ecologia e Recursos Naturais, Bloco 902, Centro de Ciências, Universidade Federal do Ceará – UFC, Campus do PICI, Av. Humberto Monte, s/n, 60455-760 Fortaleza, Ceará, Brazil; 2 Programa de Pós-Graduação em Bioprospecção Molecular, Universidade Regional do Cariri, Campus do Pimenta, Rua Cel. Antônio Luiz, 1161, Bairro do Pimenta, 63105-000, Crato, Ceará, Brazil; 3 Curso de Ciências Biológicas, Centro de Ciências do Mar, Universidade Federal do Piauí – UFPI, Campus Ministro Reis Veloso, Av. São Sebastião, 2819, Planalto Horizonte, 64202-020, Parnaíba, Piauí, Brazil

**Keywords:** Amphibians, heterogeneity, Parnaíba River delta

## Abstract

Anurans have close associations with environmental conditions and therefore represent an interesting vertebrate group for examining how resource availability and environmental variables influence species diversity. Associations between habitat heterogeneity and anuran species diversity were tested in the Restinga landscapes of the Parnaíba River delta in northeastern Brazil. Twenty-one anuran species were sampled in the rainy season during monthly excursions (December 2015 to June 2016) into areas of Restinga on two islands in the Parnaíba River delta. The fourth highest anuran diversity was found in this type of environment in Brazil and is the third in northeastern Brazil. Microenvironments, characterized by a combination of vernal pools with different vegetational and physical structures, better explained anuran species composition in the Parnaíba River delta.

## Introduction

Scientists have long attempted to explain species distribution patterns and species richness worldwide, and several ecological hypotheses and theories have been proposed (e.g., Hutchinson 1959, Pianka 1966, MacArthur and Wilson 1967, Huston 1979, Hubbell 2001, Tjørve et al. 2008), including the habitat heterogeneity hypothesis of MacArthur and MacArthur (1961), which proposed that heterogeneous environments improve species richness by allowing species coexistence.

The habitat heterogeneity hypothesis has since been used to explain distribution patterns and species richness throughout the world (e.g., Atauri and Lucio 2001, Tews et al. 2004, Bastazini et al. 2007, González-Megias et al. 2007, Vasconcelos et al. 2009, Silva et al. 2010, Jimenez-Alfaro et al. 2016). Several studies in Brazil have shown a close relationship between environmental heterogeneity and amphibian diversity, although those studies have been largely concentrated in the Amazon rain forest and Atlantic Forest (Keller et al. 2009, Vasconcelos et al. 2009, Silva et al. 2011). Studies in open formations in Brazil, such as in the morphoclimatic domains Tropical Atlantic, Caatingas, and Cerrados (see Ab’Sáber 1977 for definition of morphoclimatic domains), have been scarce (e.g., Bastazini et al. 2007, Xavier and Napoli 2011, Dória et al. 2015, respectively).

Although the habitat heterogeneity hypothesis of MacArthur and MacArthur (1961) is well understood, the measurement of this heterogeneity is difficult due to the close connection with resources variety and availability. Thus, resources such as size of water pond may be important to amphibian richness, as predicted by species-area relationship in the Islands Biogeography theory of MacArthur and Wilson (1967). In addition, duration and depth of water pond is important for amphibian reproductive success especially in regions with irregular rainfall (Becker et al. 2007).

Vegetation structure in and around water bodies is an important resource for local diversity of anurans (Bastazini et al. 2007, Dória et al. 2015) by providing conditions of more reproductive modes (Andrade et al. 2016). Amphibians are strongly influenced by environmental conditions (Duellman and Trueb 1994) and, therefore, represent an interesting vertebrate group to investigate how resource availability can influence species diversity.

The Parnaíba River delta in northeastern Brazil is dominated by Restinga coastal vegetation with sandy soils and open herbaceous, shrubby, and arboreal plant formations (Silva and Britez 2005, Santos-Filho et al. 2010, Santos-Filho et al. 2015, Serra et al. 2016) with approximately 363 known plant species belonging to 74 families (Santos-Filho et al. 2015) – indicating high local heterogeneity. The relationship of this presumed heterogeneity with anuran diversity in the Parnaiba River Delta, however, remains unknown (Andrade et al. 2016, Andrade et al. 2014, Andrade et al. 2012, Loebmann and Mai 2008). The present study aimed to test the influence of habitat heterogeneity on anurans diversity in the Restinga landscapes of the Parnaíba River Delta.

## Materials and methods

**Figure 1.**
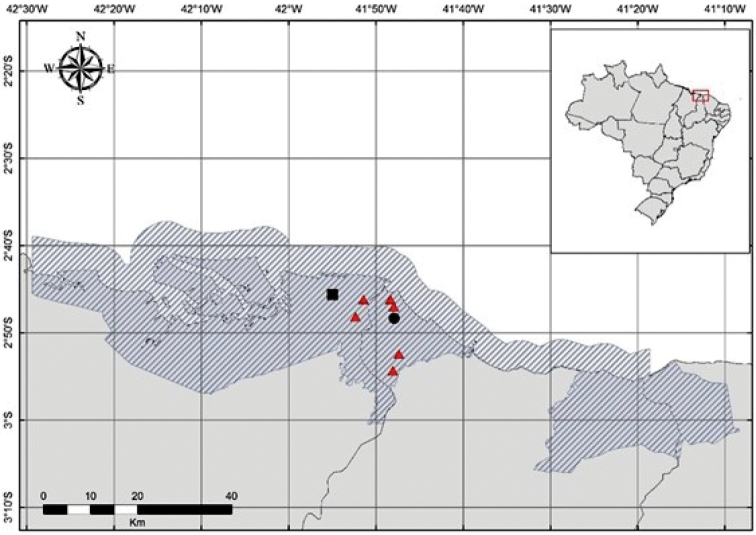
Map of the Environmental Protection Area of Parnaíba River Delta (shaded area), northeastern Brazil, with the location of the study area featuring six sampling points (red triangles). Key: black square, Canárias Island, state of Maranhão; black circle, Ilha Grande de Santa Isabel Island, state of Piauí.

Study area: The Parnaíba River Delta is contained within an Environmental Protection Area (EPA) created in August 1966, covering approximately 313,800 ha in the Brazilian states of Piauí, Maranhão, and Ceará (Fig. 1) (Brasil 2002). The region is composed of a transitional area between Caatinga and Cerrado formations and marine systems (Brasil 2002). The predominant physiognomy is the Restinga environment, quaternary habitats characterized by sandy soils with high salt concentrations covered predominantly by herbaceous and shrubby xerophytic vegetation (see Xavier et al. 2015 for the definition of a Restinga). Rainfall is concentrated mainly from January through May (IBAMA 1998).

Sampling: Amphibians were collected in areas of Restinga from two islands in the Parnaíba River Delta: Ilha Grande de Santa Isabel Island in the state of Piauí (2°52'27"S, 41°47'20"W, WGS84 datum, 5 m a.s.l.) and Canárias Island in the state of Maranhão (2°48'09"S, 41°52'19"W, WGS84 datum, 8 m a.s.l.). First we selected the areas of Restinga in Parnaíba River Delta according to the soil type (Embrapa Solos UEP Recife 2006). We then selected three Restinga landscapes covering approximately 10 km² from these areas. Using the ArcToolbox (Create Random Points) function from the software ARCGIS, version 9.3 (ESRI 2008), two plots of 1 km² were randomly chosen in each landscape as sampling points. The three Restinga landscapes and the sampling points were marked using a C7 GPS, version 1.0.

Anuran sampling was undertaken monthly on consecutive days during the rainy season (from December 2015 to June 2016) employing visual searches (Crump and Scott Jr 1994). Our sampling effort was approximately 336 hours/4 researchers. Vouchered specimens were deposited in the amphibians’ collection of the Universidade Regional do Cariri (URCA) and Universidade Federal do Piauí (UFPI) (Appendix 1). Anuran nomenclature follows Frost (2017). The species were identified according to literature and comparisons of specimens deposited in the amphibians’ collection of URCA and UFPI.

Habitat heterogeneity was quantified using seven environmental descriptors adapted from Santos et al. (2007). Values from 1 to 4 were ascribed for each environmental descriptor, being 4 the highest heterogeneity local indicator. The habitat heterogeneity of each sampling point was then quantified using the mean values of environmental descriptors (Table 1). he mean value was used to give the same importance for each environmental descriptor.

**Table 1.** Main characteristics of the six sampling points in the Parnaíba River Delta: duration (in months) of the water pond (MWP), size (in meters) of water pond (SWP), (DWP), approximate percentage of vegetation cover on water surface (PVC), types of vegetation within water (TVI), number of types of marginal vegetation (TMV) and types of margin (TM). Types of vegetation: herbaceous and macrophytes (HM), shrub (SH), and arboreal (AB). Types of margin: plans (MPMP), inclined (MI), and plan and inclined (MPI). Locality (LC) of the sampling points: Ilha Grande de Santa Isabel Island (ILG) and Canárias Island (ILC). In parentheses, the value of each environmental descriptors (1–4). Mean (Mean values of environmental descriptors).

	Point I	Point II	Point III	Point IV	Point V	Point VI
LC	ILG	ILG	ILG	ILG	ILC	ILC
MWP	5–8 (2)	5–8 (2)	5–8 (2)	1–5 (1)	1–5 (1)	1–5 (1)
SWP	300 (2)	700 (3)	400 (2)	300 (2)	300 (2)	400 (2)
DWP	> 61 (3)	> 61 (3)	> 61 (3)	31–50 (2)	31–50 (2)	> 61 (3)
PVC	76–100 (4)	76–100 (4)	31–50 (2)	31–50 (2)	31–50 (2)	31–50 (2)
TVI	HM (2)	HM (2)	HM (2)	HM (2)	HM (2)	HM (2)
TMV	AB (3)	AB (3)	AB (3)	AB (3)	AB (3)	AB (3)
TM	MPI (2)	MPI (2)	MPI (2)	MP (1)	MPI (2)	MPI (2)
Mean	2.57	2.71	2.28	1.85	2	2.14

Species distributions and associations with Brazilian morphoclimatic domains (Ab’Sáber 1977) were obtained from literature records (Bastazini et al. 2007, Valdujo et al. 2012, Roberto et al. 2013, Gondim-Silva et al. 2016). Species that occurs in the four Brazilian morphoclimatic domains were considered of wide distribution (Appendix 1).

*Statistical analyses*: the SHANNON-WIENER diversity index and EQUITY OF PIELOU (Krebs 2000) were used to measure anuran diversity; the estimator CHAO 1, which uses the number of rare species to estimate species richness of a community (Chao 1984, Colwell and Coddington 1994), was used to estimate the expected richness of amphibians. The BERGER-PARKER index (d) was used as a measure of species dominance, using Vegan package (Oksanen et al. 2016). We then produced sample-based accumulation curves with 1000 sampling randomizations, using ESTIMATE S VERSION 9.1 software (Colwell 2013) to verify if the sampling effort was sufficient to adequately represent the species community.

The normal distribution assumption was tested for both diversity and habitat heterogeneity data using the SHAPIRO-WILK test (Shapiro and Wilk 1965), at each sampling point, and was not rejected (diversity p-value = 0.5653 and habitat heterogeneity p-value = 0.8006). A linear regression analysis was used to test the influence of habitat heterogeneity (independent variable) on anurans diversity (dependent variable) (null hypothesis of no association between anuran diversity and habitat heterogeneity). All statistical analyses were performed in R software (R Development Core Team 2011), using Vegan package (Oksanen et al. 2016).

## Results

1822 anuran specimens were recorded, belonging to six families (Bufonidae, Hylidae, Leptodactylidae, Microhylidae, Odontophrynidae, and Phyllomedusidae), 12 genera, and 21 species (see Appendix 1 and 2).

**Figure 2.**
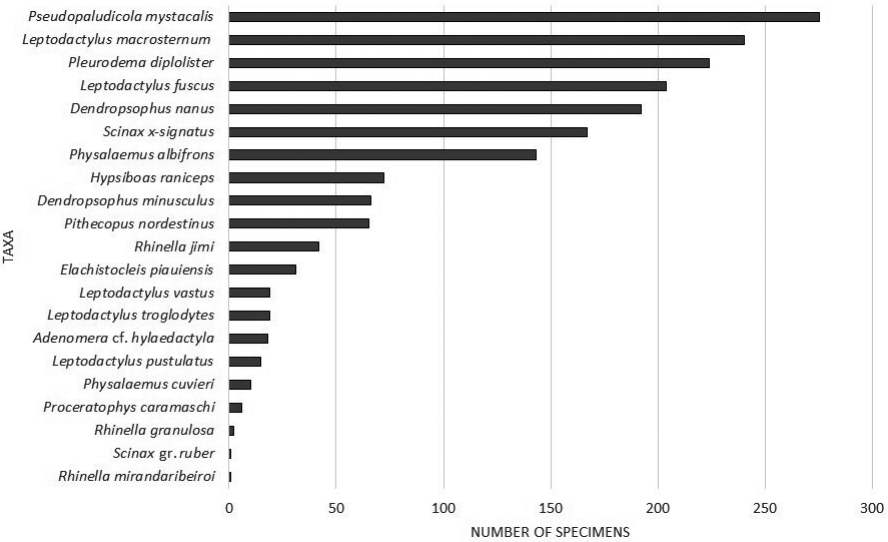
Abundance of anurans species obtained in Ilha Grande de Santa Isabel Island and Canárias Island, Parnaíba River Delta, Northeastern Brazil.

The most abundant species belonged to the families Leptodactylidae and Hylidae (Fig. 2), and they also showed the highest BERGER-PARKER dominance values (d): *Pseudopaludicola mystacalis* (d = 0.14), *Leptodactylus macrosternum* (d = 0.13), *Pleurodema diplolister* (d = 0.12), *Leptodactylus fuscus* (d = 0.11), and *Dendropsophus nanus* (d = 0.10). The CHAO 1 species richness estimator was 21.5 ± 3 species in the Parnaíba River Delta; 18 ± 1 species in Ilha Grande de Santa Isabel Island and 14 ± 3 species in Canárias Island (Tab. 2).

**Figure 3.**
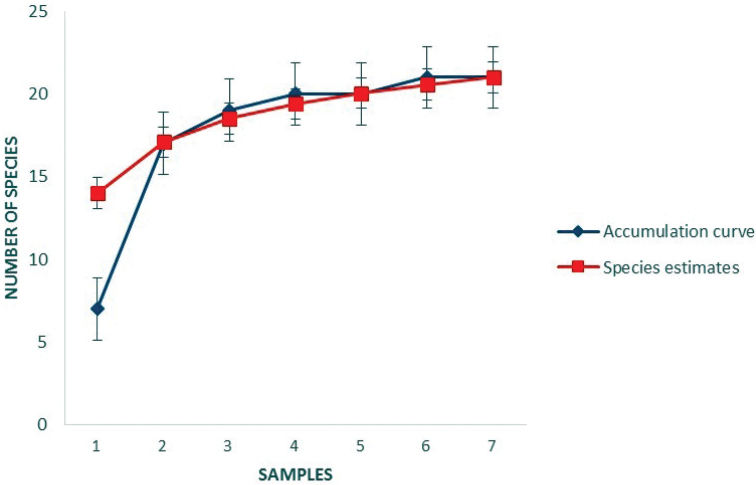
Accumulation curve for anurans sampled in the Parnaíba River Delta, northeastern Brazil, constructed from 1000 randomizations on the order of samplings. Species estimates (Chao 1 estimator).

The sample-based accumulation curve tended asymptote (Fig. 3), which suggest that the sampling effort was sufficient to adequately represent the species community in Parnaíba River Delta, northeastern Brazil.

**Table 2.** Anuran diversity in the Parnaíba River Delta (PRD), Ilha Grande de Santa Isabel Island (ILG) and Canárias Island (ILC), with data on species richness (CHAO 1 species richness estimator), dominant species (BERGER-PARKER index) and evenness (PIELOU’s index J’).

	PRD	ILG	ILC
Number of individuals	1822	1465	357
Species richness (observed)	21	18	14
Species richness (estimated)	21.5 ± 3	18 ± 1	14 ± 3
Dominant species	*P. mystacalis*	*P. mystacalis*	*L. fuscus*
Dominance observed	14%	14%	19%
Shannon - Wiener (H’)	2.485	2.476	2.185
Pielou’s index J’	0.8165	0.8569	0.8282

**Table 3.** Habitat heterogeneity, SHANNON-WIENER diversity index, evenness (Pielou’s index J’) and habitat heterogeneity value for each sampling point in the Parnaíba River Delta.

Sampled points	Diversity index	Pielou’s index J’	Heterogeneity
Point I	H’ = 2.279	J’ = 0.8637	He = 2.57
Point II	H’ = 2.467	J’ = 0.8708	He = 2.71
Point III	H’ = 2.220	J’ = 0.8935	He = 2.28
Point IV	H’ = 1.768	J’ = 0.8502	He = 1.85
Point V	H’ = 1.815	J’ = 0.7881	He = 2
Point VI	H’ = 2.052	J’ = 0.8557	He = 2.14

The species richness at the six sampling points varied from 8 to 17 (Tab. 2). The highest values of species diversity were recorded at points II, I and III, respectively, while point IV had the lowest diversity value. The highest values of habitat heterogeneity were observed at points II, I, and III, respectively, all located in Ilha Grande de Santa Isabel Island. Points V and VI showed intermediated values, while point IV had the lowest habitat heterogeneity value (Tab. 3). The combination of all environmental descriptors is the reason for different heterogeneity indexes in present study.

**Figure 4.**
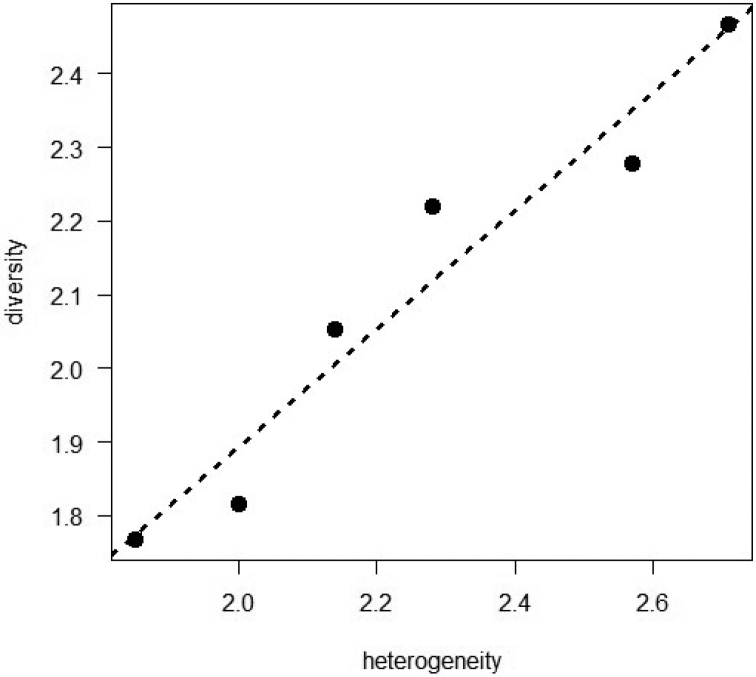
Association between anurans’ species diversity (SHANNON-WIENER diversity index) and habitat heterogeneity in the Parnaíba River Delta, Northeastern Brazil (R² = 0.9204, p-value = 0.0015). Computation of the habitat heterogeneity index is explained in Material and methods.

The linear regression analysis evidenced that the habitat heterogeneity of the Restinga environment in the Parnaíba River Delta is able to explain the anuran diversity (R² = 0.9204, p = 0.0015) (Fig. 4).

## Discussion

The Restinga of the Parnaíba River Delta have the fourth highest anuran richness in this type of environment in Brazil and the third in northeastern Brazil (21 species). The Restinga areas with the highest anuran diversity were encountered in the municipalities of Mata de São João (34 species; Bastazini et al. 2007, Oliveira and Rocha 2015, Xavier et al. 2015) and Conde (33 species; Gondim-Silva et al. 2016), both in the state of Bahia, and in the municipality of Grumari, Rio de Janeiro state (22 species; Telles et al. 2012).

The anuran species composition of the Parnaíba River Delta was similar to that reported by Borges-Leite et al. (2014) and Gondim-Silva et al. (2016) for the municipalities of São Gonçalo do Amarante and Conde, in the Brazilian states of Ceará and Bahia, respectively. The aforementioned study in the Ceará state was carried out in an ecotonal environment with floristic elements of Caatinga, Cerrado and Restinga (Borges-Leite et al. 2014) and the study in Bahia state included only “Open Restinga” (Gondim-Silva et al. 2016). The similarity between the present work and these studies could be explained by the presence of floristic elements of Caatinga, Cerrado and Restinga in our open Restinga area studied.

Nevertheless the Restinga of the Parnaíba River Delta differed greatly from Restinga sites in southeastern Brazil (states of São Paulo, Rio de Janeiro and Espírito Santo; Rocha et al. 2008, Silva et al. 2008, Vilela et al. 2011, Telles et al. 2012) and other regions of the state of Bahia (municipalities of Prado, Trancoso and Mata de São João; Bastazini et al. 2007, Rocha et al. 2008, Narvaes et al. 2009). These studies included lowland forests that can be very important for explaining the differences in anuran composition between them. The high habitat heterogeneity in Restinga environments (Gomes et al. 2016), however, could also account for those differences.

Increased habitat structural complexity results in greater species diversity (MacArthur and MacArthur 1961), with homogeneous areas showing less microhabitat availability, which hampers species coexistence and resource partitioning (Macarthur and Levins 1967). Highly heterogeneous environments promote higher species richness by promoting the coexistence, persistence, and diversification of species at different spatial and temporal scales (Stein and Kreft 2014).

Positive relationships between habitat heterogeneity and anuran diversity have been recorded in different morphoclimatic domains in Brazil, as well in the present study. Habitat heterogeneity has been shown to influence anuran diversity in Restinga areas in northeastern Brazil (Bastazini et al. 2007), in “Campo rupestre” vegetation in the Caatinga (Xavier and Napoli 2011), and in Cerrado vegetation with a predominance of semi-deciduous seasonal forest (Dória et al. 2015). A clear relationship between habitat heterogeneity and anuran diversity was recorded in the Atlantic Forest (Lop et al. 2012, Santos et al. 2012) as did Silva et al. (2011) in pasture areas, both in southeastern Brazil. Some studies, however, could not identify relationships between habitat heterogeneity and anuran diversity (Eterovick 2003, Vasconcelos and Rossa-Feres 2005, Santos et al. 2007), and more studies will consequently be necessary to elucidate the importance of environment heterogeneity to species diversity.

Anuran populations from the Restinga of the Parnaíba River Delta are influenced by habitat complexity and the variety of available microhabitats, in agreement with Bastazini et al. (2007) who highlighted the importance of shrub formations and bromeliad densities to explain changes in anuran composition in Restinga environments.

Earlier studies highlighted the importance of pond size and edge vegetation to anuran diversity (Parris and McCarthy 1999, Burne and Griffin 2005, Bastazini et al. 2007, Vieira et al. 2007, Xavier and Napoli 2011, Dória et al. 2015, Gonçalves et al. 2015). Furthermore, microenvironments composed of vernal pools with different edge vegetation structures and percentage of vegetation cover on water surface better explained the different compositions of anuran communities in Parnaíba River Delta.

## Appendix 1

Anuran species obtained at the Restinga of the Parnaíba River delta, northeastern Brazil. Morphoclimatic domains (Ab’Sáber 1977): Caatingas (CA), Cerrados (CE), Tropical Atlantic (AT), and Equatorial Amazonian (AM). Species that occur in the four Brazilian morphoclimatic domains were considered as having a wide distribution (WD). Species without voucher specimens were represented by photography.

**Table:**

Taxon	Voucher specimens	Sampling points (I–VI)	Morphoclimatic domains
**Bufonidae**			
*Rhinella granulosa* (Spix, 1824)	Photographed	I, II	CA, AT
*Rhinella jimi* (Stevaux, 2002)	Photographed	All sampling points	CA, AT
*Rhinella mirandaribeiroi* (Gallardo, 1965)	Photographed	V	CE
**Hylidae**			
*Dendropsophus minusculus* (Rivero, 1971)	URCA-H12120	I, II, V, VI	WD
*Dendropsophus nanus* (Boulenger, 1889)	CZDP473	I, II, III, V, VI	WD
*Hypsiboas raniceps* (Cope, 1862)	URCA-H12115	I, II, III, V	WD
*Scinax* sp. (gr. ruber)	URCA-H12123	II	No information
*Scinax x-signatus* (Spix, 1824)	Photographed	All sampling points	WD
**Leptodactylidae**			
Adenomera cf. hylaedactyla (Cope, 1868)	URCA-H12125	V	AM, CE, AT
*Leptodactylus fuscus* (Schneider, 1799)	Photographed	All sampling points	WD
*Leptodactylus macrosternum* Miranda-Ribeiro, 1926	Photographed	All sampling points	WD
*Leptodactylus pustulatus* (Peters, 1870)	URCA-H12126	II	CE
*Leptodactylus troglodytes* Lutz, 1926	CZDP485	II, IV	CA, CE, AT
*Leptodactylus vastus* Lutz, 1930	Photographed	I, II, III	CA, CE, AT
*Physalaemus albifrons* Spix, 1824	Photographed	I, II, III, IV, VI	CA, CE, AT
*Physalaemus cuvieri* Fitzinger, 1826	CZDP470	II	WD
*Pleurodema diplolister* Peters, 1870	Photographed	I, II, III, IV, VI	CA, CE, AT
*Pseudopaludicola mystacalis* (Cope, 1887)	URCA-H12118	I, II, III, V	WD
**Microhylidae**			
*Elachistocleis piauiensis* Caramaschi and Jim, 1983	URCA-H12124	I, III, VI	CA, CE
**Odontophrynidae**			
*Proceratophrys caramaschii* Cruz, Nunes and Juncá, 2012	Photographed	VI	CA
**Phyllomedusidae**			
*Pithecopus nordestinus* (Caramaschi, 2006)	Photographed	I, II, III, IV	CA, CE, AT

## Appendix 2

Anurans recorded at the Restinga of the Parnaíba River Delta, Northeastern Brazil. In brackets, the vouchered specimen with the acronym of the scientific collection followed by the respective institutional registration number and specimen snout-vent length (SVL) in millimeters. Some species only have photographic records. (A) *Rhinella granulosa*, (B) *R. jimi*, (C) *R. mirandaribeiroi*, (D) *Dendropsophus minusculus* (URCA-H12120, SVL 18.4), (E) *D. nanus* (CZDP473, SVL 19.2), (F) *Hypsiboas raniceps* (URCA-H12115, SVL 62.6), (G) *Scinax* sp. (gr. ruber) (URCA-H12123, SVL 20.1), (H) *S. x-signatus*, (I) Adenomera cf. hylaedactyla (URCA-H12125, SVL 15.8), (J) *Leptodactylus fuscus*, (K) *L. macrosternum*, (L) *L. pustulatus* (URCA-H12126, SVL 41.2), (M) *L. troglodytes* (CZDP485, SVL 43.3), (N) *L. vastus*, (O) *Physalaemus albifrons*, (P) *P. cuvieri* (CZDP470, SVL 24.5), (Q) *Pleurodema diplolister*, (R) *Pseudopaludicola mystacalis* (URCA-H12118, SVL 11.9), (S) *Elachistocleis piauiensis* (URCA-H12124, SVL 30.2), (T) *Proceratophrys caramaschii*, (U) *Pithecopus nordestinus*. Photographs: Kássio C. Araújo and Ocivana A. Pereira.
